# Non-insufflation endoscopic intermuscular dissection in the management of a rectal neuroendocrine tumor

**DOI:** 10.1055/a-2616-6799

**Published:** 2025-07-01

**Authors:** Xiangji Liu, Jingjing Yao, Jing Wang, Taiping Wang, Haiyan Zhang, Ling Wang, Jindong Fu

**Affiliations:** 1 549615 Department of Gastroenterology, Rizhao Peopleʼs Hospital, Rizhao, China; 2 549615 Department of Pathology, Rizhao Peopleʼs Hospital, Rizhao, China

**Fig. 1:**
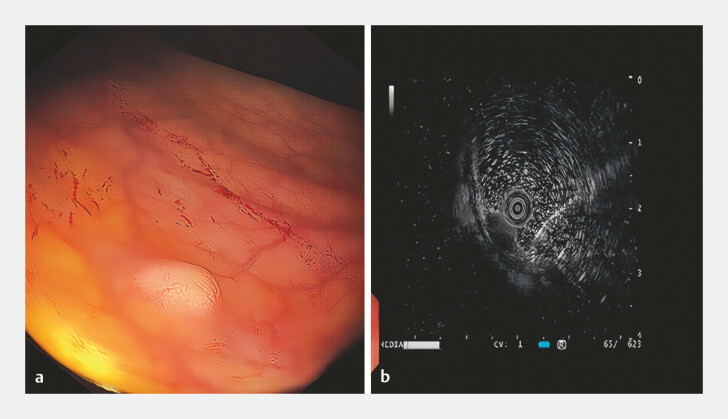
Endoscopic images of the lesion. **a** Enteroscopic. **b** Ultrasonographic.

A 42-year-old male was identified with a submucosal lesion in the rectum during a routine colonoscopy ( Fig. 1 **a** ). Endoscopic ultrasound confirmed that the lesion, approximately 8 mm in size with a yellowish appearance, was located in the deep mucosal and submucosal layers ( Fig. 1 **b** ). A neuroendocrine tumor (NET) was highly suspected. Endoscopic resection was requested.

**Fig. 2:**
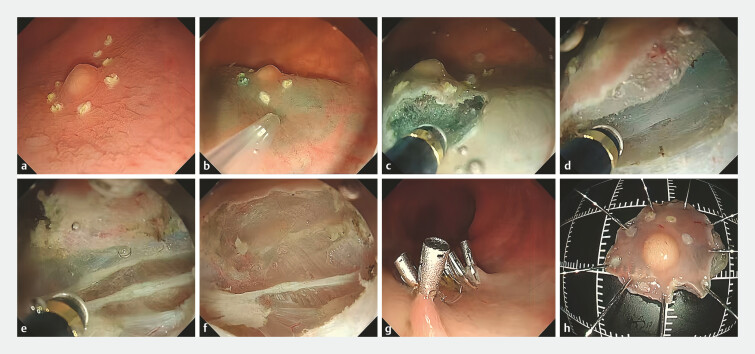
Images of underwater endoscopic intermuscular dissection procedure. **a** Marking around lesion underwater. **b** Submucosal injection underwater. **c** Incising the anal side of the lesion underwater. **d** Stripping the submucosal layer and exposing the muscle layer. **e** Incision of circular muscle layer, exposure of longitudinal muscle, and opening of the intermuscular space. **f** Stripped wound showing intact longitudinal muscle. **g** Metal clips for wound closure. **h** Postoperative specimen.

Endoscopic intermuscular dissection (EID) has recently emerged as a new endoscopic technique for NETs due to its efficacy in reducing positive vertical margins 1 2 . Building on this, we adopted a new modified method for lesion resection. The rectal cavity was filled with saline solution, submerging the entire lesion. Routine marking ( Fig. 2 **a** ), submucosal injection ( Fig. 2 **b** ), and circumferential incision procedures ( Fig. 2 **c** ) all were performed underwater ( Video 1 ). After submucosal dissection to expose the muscle layer ( Fig. 2 **d** ), the circular muscle was incised ( Fig. 2 **e** ) and the longitudinal muscular layer was exposed. With the assistance of a transparent cap, the intermuscular space was accessed. Dissection within the intermuscular space was continued until the tumor was resected ( Fig. 2 **f** , Fig. 2 **h** ). The wound was closed with metal clips ( Fig. 2 **g** ). The entire procedure was performed underwater without insufflation. The patient was placed on a 48-hour fast following the procedure and was discharged in good condition 3 days later. Postoperative pathology confirmed a well differentiated NET (grade 1), with negative horizontal and vertical margins.

Non-insufflation endoscopic intermuscular dissection in management of a rectal neuroendocrine tumor.Video 1

This modified method can enhance procedure efficacy because it offers the following advantages. First, the entire procedure was performed underwater, avoiding abdominal discomfort caused by insufflation during surgery. Second, a clear field of vision can be maintained throughout 3 . Third, there is minimal traction force underwater, making the intermuscular space easier to expose and avoiding damage to the longitudinal muscular layer. Last, traction force on the wound is minimal underwater, reducing tension for suturing and making wound closure easier.
